# The *Agrobacterium tumefaciens* Ti Plasmid Virulence Gene *virE2* Reduces *Sri Lankan Cassava Mosaic Virus* Infection in Transgenic *Nicotiana benthamiana* Plants

**DOI:** 10.3390/v7052641

**Published:** 2015-05-22

**Authors:** Thulasi Raveendrannair Resmi, Thomas Hohn, Barbara Hohn, Karuppannan Veluthambi

**Affiliations:** 1 Department of Plant Biotechnology, School of Biotechnology, Madurai Kamaraj University, Madurai, Tamil Nadu 625021, India; E-Mail: trresmi@gmail.com; 2 Institute of Botany, University of Basel, Schoenbeinstrasse 6, 4056 Basel, Switzerland; E-Mail: thomas.hohn@fmi.ch; 3 Friedrich Miescher Institute for Biomedical Research, 4058 Basel, Switzerland; E-Mail: barbara.hohn@fmi.ch

**Keywords:** geminivirus, agroinfection, ssDNA binding, cassava mosaic virus, SLCMV

## Abstract

Cassava mosaic disease is a major constraint to cassava cultivation worldwide. In India, the disease is caused by *Indian cassava mosaic virus* (ICMV) and *Sri Lankan cassava mosaic virus* (SLCMV). The *Agrobacterium* Ti plasmid virulence gene *virE2*, encoding a nuclear-localized, single-stranded DNA binding protein, was introduced into *Nicotiana benthamiana* to develop tolerance against SLCMV. Leaf discs of transgenic *N. benthamiana* plants, harboring the *virE2* gene, complemented a *virE2* mutation in *A. tumefaciens* and produced tumours. Three tested *virE2* transgenic plants displayed reduction in disease symptoms upon agroinoculation with SLCMV DNA A and DNA B partial dimers. A pronounced reduction in viral DNA accumulation was observed in all three *virE2* transgenic plants. Thus, *virE2* is an effective candidate gene to develop tolerance against the cassava mosaic disease and possibly other DNA virus diseases.

## 1. Introduction

Geminiviruses constitute a large family of plant viruses which infect a wide range of crops and cause enormous losses worldwide. They possess monopartite or bipartite genomes, comprising circular, single-stranded DNAs and are encapsidated in paired icosahedral particles [1,2]. The family *Geminiviridae* is classified into seven genera, *Begomovirus*, *Mastrevirus*, *Curtovirus*, *Becurtovirus*, *Eragrovirus*, *Topocuvirus* and *Turncurtovirus* [3].

Cassava is a major food and commercial crop in Africa and India and cassava mosaic disease is the major constraint to cassava cultivation [4,5]. The disease is caused in India by *Indian cassava mosaic virus* (ICMV) and *Sri Lankan cassava mosaic virus* (SLCMV) [6,7]. Transgenic expression of pathogen-derived genes is widely used to obtain geminivirus resistance in crop plants [8]. Strategies of engineering viral resistance by expressing full-length or truncated viral proteins such as replication associated protein, coat protein and movement protein are effective in controlling viral infection in model as well as crop plants [9,10,11,12,13,14]. Gene silencing strategies using sense RNA, anti-sense RNA and double-stranded RNA have been successfully used to generate geminivirus resistance [15,16,17,18,19].

There are fewer examples with respect to the use of non-viral protein genes to develop virus resistance. Padidam *et al.* [20] reported that M13 bacteriophage gene 5 protein, which binds ssDNA, was effective in restricting *Tomato leaf curl virus* infections. Sera [21] reported that expression of an artificial zinc finger protein targeting the replication origin of *Beet severe curly top virus* efficiently blocked viral DNA replication in transgenic *Arabidopsis* plants. Sunitha *et al.* [22] expressed the *A. tumefaciens* Ti plasmid *virE2* gene in tobacco and showed a reduction of viral DNA accumulation in leaf discs agroinoculated with *Mungbean yellow mosaic virus* (MYMV) partial dimers. Here, we report the efficacy of the *virE2* gene in restricting SLCMV infection in transgenic *Nicotiana benthamiana* plants. This report shows that the *virE2* gene is a good non-viral gene that can be used to develop broad spectrum geminivirus tolerance.

## 2. Materials and Methods

### 2.1. Plasmid Constructs

Construction of pCAM-*virE2*: The *Agrobacterium tumefaciens virE2* gene with an intron, under the transcriptional control of CaMV 35S promoter, was cloned as a *Bam*HI/*Pst*I fragment in the corresponding sites of pCAMBIA1380 to yield pCAM-*virE2*. pCAM-*virE2* was mobilized into the *A. tumefaciens* strain GV3101 which has the disarmed vector pPM6000 [22].

### 2.2. Viral Clones

SLCMV-[Attur2] DNA A (NCBI Accession No. KP455484) and DNA B (NCBI Accession No. 455485) were cloned following rolling circle amplification (GE Healthcare UK Ltd., Little Chalfont, UK) from field-infected cassava leaf samples.

### 2.3. Construction of Partial Dimers of SLCMV-[Attur2] DNA A and DNA B

The plasmid pBS-SLCMV-At-A harbors the full length SLCMV-[Attur2] DNA A (2758 bp) as a *Pst*I fragment in pBSIIKS^+^. An 1.7-kb *Pst*I/*Hin*dIII fragment of DNA A from pBS-SLCMV-At-A was cloned into pBSIIKS^+^ to yield pBS-SLCMV-At0.6A. The full length DNA A, as a *Pst*I fragment from pBS-SLCMV-At-A, was cloned into the *Pst*I site of pBS-SLCMV-At0.6A to yield the partial dimer clone of SLCMV-[Attur2] DNA A in pBSIIKS^+^ (pBS-SLCMV-At1.6A). A *Sac*I/*Sal*I fragment from pBS-SLCMV-At1.6A comprising the partial dimer was cloned into the corresponding sites of the binary vector pPZP201 [23] to yield pPZP-SLCMV-At1.6A.

The plasmid pBS-SLCMV-At-B harbors the full-length SLCMV-[Attur2] DNA B (2738 bp) in pBSIIKS^+^ as a *Bam*HI fragment. A 2.3-kb *Bam*HI/*Kpn*I fragment from pBS-SLCMV-At-B was inserted into the corresponding sites of the binary vector pPZP201 to yield pPZP-SLCMV-At0.8B. The full length SLCMV-[Attur2] DNA B, as a *Bam*HI fragment from pBS-SLCMV-At-B, was cloned in the *Bam*HI site of pPZP-SLCMV-At0.8B to yield pPZP-SLCMV-At1.8B with the partial dimer of SLCMV-[Attur2] DNA B.

pPZP-SLCMV-At1.6A and pPZP-SLCMV-At1.8B with the DNA A and DNA B partial dimers, respectively, were introduced independently into the *A. tumefaciens* strain Ach5 by triparental mating [24] or electroporation (Bio-Rad, Hercules, CA, USA) and the transconjugants or transformants were confirmed by Southern blot analysis.

### 2.4. N. benthamiana Transformation

Leaf discs from axenically-grown *N. benthamiana* plants were transformed with *A. tumefaciens* (pCAM-*virE2*) as described by Horsch *et al.* [25]. Transgenic shoots were selected on Murashige and Skoog (MS) [26] shoot induction medium containing 4 µM 6-benzylaminopurine, 0.5 µM α-naphthaleneacetic acid, 50 mg/L hygromycin and 250 mg/L cefotaxime. The shoots were subjected to root induction for two days in half-strength liquid MS medium containing 20 µM indolebutyric acid. The shoots were then kept on half-strength solid MS basal medium containing 25 mg/L hygromycin, 250 mg/L cefotaxime and 150 mg/L timentin for root development.

### 2.5. Southern Blot Analysis

Total plant DNA was extracted [27] and estimated by fluorometry. DNA was digested with suitable restriction enzymes and electrophoresed in a 0.8% (w/v) agarose gel in 1 X TNE (40 mM Tris-acetate, pH 7.5, 20 mM sodium acetate, 2 mM EDTA) or 1 X TBE (Tris-borate-EDTA) buffers. DNA was transferred [28] to the Zeta-probe nylon membrane (Bio-Rad, Hercules, CA, USA). DNA was labeled with [α-^32^P]dCTP using the Megaprime DNA labeling system (GE Healthcare UK Ltd., Little Chalfont, UK) to prepare probes. Hybridizations were carried out at 65°C and high stringency post hybridization washes were performed [29].

### 2.6. VirE2 Complementation Assay

Control *N. benthamiana* leaf discs were infected with wild type *A. tumefaciens* strain A348 (with the wild type Ti plasmid pTiA6) and the mutant *A. tumefaciens* strain A348mx358 which harbors pTi358 with Tn*3* HoHo1 insertion in the *virE2* gene [30]. For VirE2 complementation assay, leaf discs from 3-week-old control or *virE2* transgenic *N. benthamiana* plants were preincubated for two days on MS shoot induction medium and infected with *A. tumefaciens* strains grown to A_600_ = 1 in AB minimal medium [31]. The leaf discs were placed on MS shoot induction medium for co-cultivation. After two days, the leaf discs were transferred to a hormone-free MS medium with 250 mg/L cefotaxime.

### 2.7. Northern Blot Analysis

Young leaf tissue (0.5 g) of *N. benthamiana* plants was used for RNA extraction. Total RNA was extracted using the Trizol method as per instructions provided by the manufacturer (Sigma-Aldrich, St. Louis, MO, USA). RNA was estimated in a spectrophotometer. Total RNA (10 µg) was electrophoresed in a 1.2% (w/v) agarose gel containing 1% formaldehyde (v/v) in 1 × MOPS (3-(N-morpholino)propane sulphonic acid) buffer. Northern blot analysis was performed as described by Pawlowski *et al.* [32].

### 2.8. Agroinfection

Agroinfection of 3-week-old *N. benthamiana* plants was performed with the *A. tumefaciens* strain Ach5 harboring the partial dimers of SLCMV-[Attur2] DNA A and DNA B (two strain method). The *A. tumefaciens* (pPZP-SLCMV-At1.6A) and *A. tumefaciens* (pPZP-SLCMV-At1.8B) cultures were grown in AB minimal medium to A_600_ = 1 and cells were centrifuged at 28 °C at 1100× *g*. The pellets were resuspended in AB minimal medium (pH 5.6) containing 100 µM acetosyringone. The cultures were mixed (1:1) and agroinfection was performed by inoculating 10 µL of the bacterial mixture above the node of the first fully expanded leaf from the top. The stem was immediately pricked with a 30 G needle [33,34].

### 2.9. Densitometry Analysis

Integrated density values (IDV) of the autoradiogram were determined by using the AlphaEase™ software (Version 5.5, Alpha Innotech Corpoartion, San Leandro, CA, USA).

## 3. Results

### 3.1. Characterization of N. benthamiana Plants Transformed with the A. tumefaciens virE2 Gene

**Figure 1:**
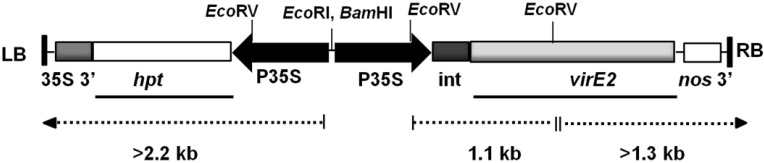
T-DNA of the binary vector pCAM-*virE2* [22]. LB, left T-DNA border; RB, right T-DNA border; P35S, *Cauliflower mosaic virus* (CaMV) 35S promoter; 35S 3’, CaMV 35S polyadenylation signal; *hpt*, hygromycin phosphotransferase gene; int, intron; nos3’, nopaline synthase polyadenylaton signal. Junction fragments (>2.2 kb and >1.3 kb) and internal T-DNA fragment (1.1 kb) are shown in dotted lines. Bold lines represent the region of *hpt* and *virE2* genes which were used as probes.

The binary plasmid pCAM-*virE2* (Figure 1), which harbors the *virE2* gene of the octopine type Ti plasmid under the transcriptional control of the CaMV 35S promoter [22], was used to transform *N. benthamiana* leaf discs. pCAM-*virE2* contains the hygromycin phosphotransferase gene (*hpt*) as plant selectable marker.

**Figure 2:**
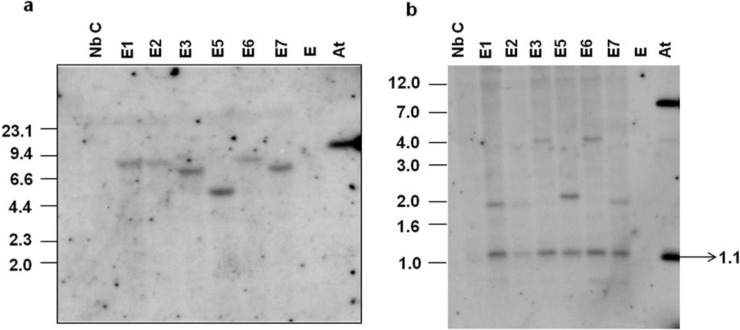
(**a**) Southern blot analysis of *virE2* transgenic *Nicotiana benthamiana* plants with the *hpt* probe. Total DNA (10 µg) from transgenic (E1-E7) as well as control (Nb C) plants was digested with *Eco*RI. *Agrobacterium tumefaciens* (pCAM-*virE2*) DNA (0.25 ng) digested with *Eco*RI (At) was used as a positive control. The *hpt* gene (50 ng) labeled with [α-^32^P]dCTP was used as the probe; (**b**) Internal T-DNA fragment and junction fragment analyses of *virE2* transgenic plants by Southern blotting. Total DNA (10 µg) from transgenic (E1-E7) and control (Nb C) plants was digested with *Eco*RV. Total *A. tumefaciens* (pCAM-*virE2*) DNA (0.25 ng) digested with *Eco*RV served as a positive control (At). The *virE2* gene (50 ng) labelled with [α-^32^P]dCTP was used as the probe.

Six hygromycin-resistant plants, selected on 50 mg/L hygromycin, were analyzed by Southern blotting for T-DNA integration. DNA from the plants E1, E2, E3, E5, E6 and E7 was digested with *Eco*RI and the blot was probed with the *hpt* gene. Junction fragments longer than 2.2 kb were expected to hybridize (Figure 1). All six plants displayed single junction fragments (Figure 2a) indicating that all six are single-copy transgenic plants. The plants E1, E2 and E7 exhibited junction fragments of similar size and thus could have regenerated from the same transformed callus. To address this possibility and to confirm the presence of the *virE2* gene in all transgenic plants, a Southern blot analysis was performed by digesting the DNA with *Eco*RV and hybridizing the blot with the *virE2* probe. An internal T-DNA fragment of 1.1 kb and a junction fragment longer than 1.3 kb are expected to hybridize (Figure 1). All plants showed expected hybridization of the internal T-DNA fragment of 1.1 kb comprising *virE2* (Figure 2b). Plants E1, E2 and E7 displayed hybridization to junction fragments of the same size (2.0 kb). Thus, the plants E1, E2 and E7 were inferred as single- copy transgenic plants which regenerated from the same transformed callus. The remaining transgenic plants E3, E5 and E6 showed individual single junction fragments confirming that all are single-copy transgenic plants.

### 3.2. Complementation of the A. tumefaciens virE2 Mutation by VirE2 Expressed in Transgenic N. benthamiana Plants

**Figure 3:**
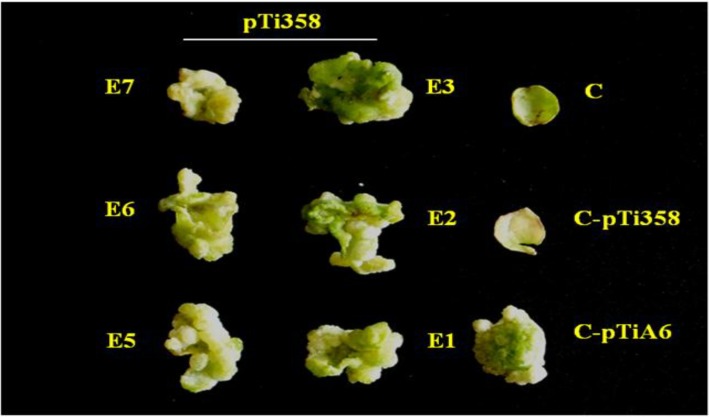
Functional complementation analysis of *virE2* mutation in the *Agrobacterium tumefaciens* strain A348mx358 in leaf discs of *virE2* transgenic plants grown on MS medium without hormones. *A. tumefaciens* A348mx358 which harbors pTi358 with *virE2* mutation [30] was used to infect the leaf discs of untransformed *N. benthamiana* control plants (C-pTi358) and the *virE2* transgenic plants (E1-E7). Untransformed control leaf discs infected with *A. tumefaciens* (pTiA6) (C-pTiA6) was used as the positive control. C: untransformed, uninfected *N. benthamiana* leaf disc.

The function of the VirE2 protein in the transgenic *N. benthamiana* plants was evaluated by its ability to complement the *virE2* mutation of the *A. tumefaciens* strain A348mx358 [35]. Leaf discs of untransformed, control *N. benthamiana* plants infected with the wild type *A. tumefaciens* A348 harboring pTiA6 formed tumours when kept on hormone-free MS medium (Figure 3). Leaf discs of untransformed *N. benthamiana* plants, infected with the *virE2* mutant *A. tumefaciens* (pTi358), did not form tumors. Leaf discs of all six *virE2* transgenic *N. benthamiana* plants, E1, E2, E3, E5, E6 and E7 infected with the *virE2* mutant *A. tumefaciens* strain A348mx358, efficiently formed tumors on hormone-free MS medium (Figure 3). Thus, the transgenically expressed VirE2 complemented the *virE2* mutation of the *A. tumefaciens* strain A348mx358. The results showed that the plant-expressed VirE2 protein is functional.

**Figure 4:**
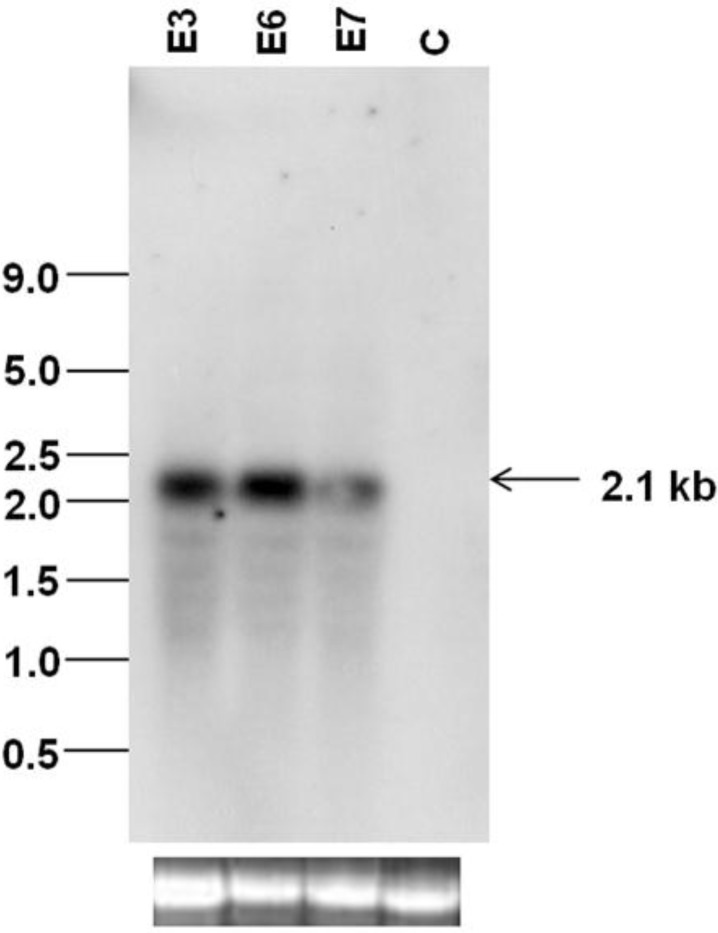
Northern blot analysis of the *virE2* transgenic plants (E3, E6, E7) with the *virE2* probe. Total RNA from untransformed *Nicotiana benthamiana* was used as the negative control (C). The bottom panel represents equal loading of RNA (10 µg) in all lanes.

Of the four independent transgenic plants E3, E5, E6 and E7, the plant E5 did not set seeds. Therefore, the plants E3, E6 and E7 were forwarded to T_1_ generation and the T_1_ plants (*virE2* PCR-positive) were used for northern blot analysis and SLCMV infection analysis. Northern blotting with the *virE2* probe showed that all the three transgenic plants E3, E6 and E7 accumulated the 2.1-kb *virE2* transcript (Figure 4).

### 3.3. SLCMV-Infected N. benthamiana Plants Displayed a Reduction in Viral Disease Symptoms and Viral DNA Level

**Figure 5:**
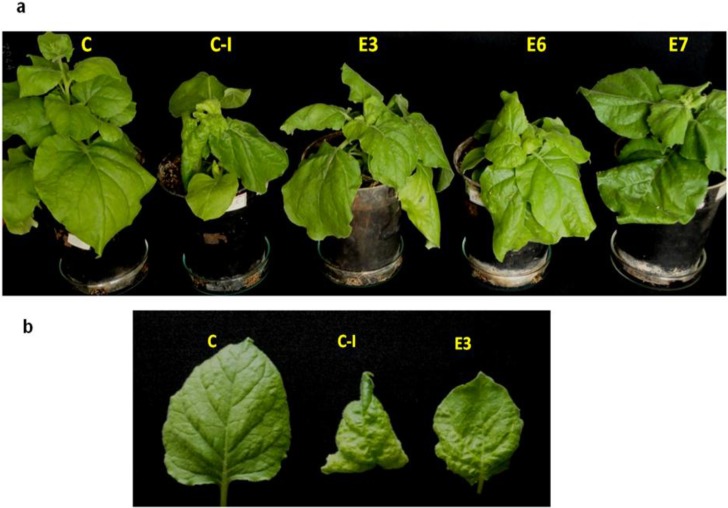
(**a**) Viral symptoms in the *virE2* transgenic *Nicotiana benthamiana* plants (E3, E6, E7) agroinoculated with the partial dimers of Sri Lankan cassava mosaic virus (SLCMV)-[Attur2] DNA A and DNA B. C: untransformed, uninfected *N. benthamiana* plant. C-I: agroinoculated control *N. benthamiana* plant as the positive control; (**b**) Individual leaves from uninfected control (C), infected control (C-I) and *virE2* transformed (E3) *N. benthamiana* plants.

The effect of VirE2 expression in transgenic *N. benthamiana* plants E3, E6 and E7 on SLCMV disease symptoms and viral DNA accumulation was studied. T_1_ plants were raised and the transgenic T_1_ plants were identified by PCR with the *virE2* primers (data not shown). The transgenic plants were phenotypically similar to the control *N. benthamiana* plants. Three-week-old *virE2* transgenic and control plants were agroinoculated with the *A. tumefaciens* strain Ach5 harboring the partial dimers of SLCMV-[Attur2] DNA A and DNA B. Two weeks post-infection, control plants infected with partial dimers of SLCMV showed severe stunting and downward leaf curling. These symptoms were noticeably reduced in the E3, E6 and E7 transgenic plants (Figure 5a,b). A discrete reduction in downward leaf curling was observed in all the transgenic plants.

**Figure 6:**
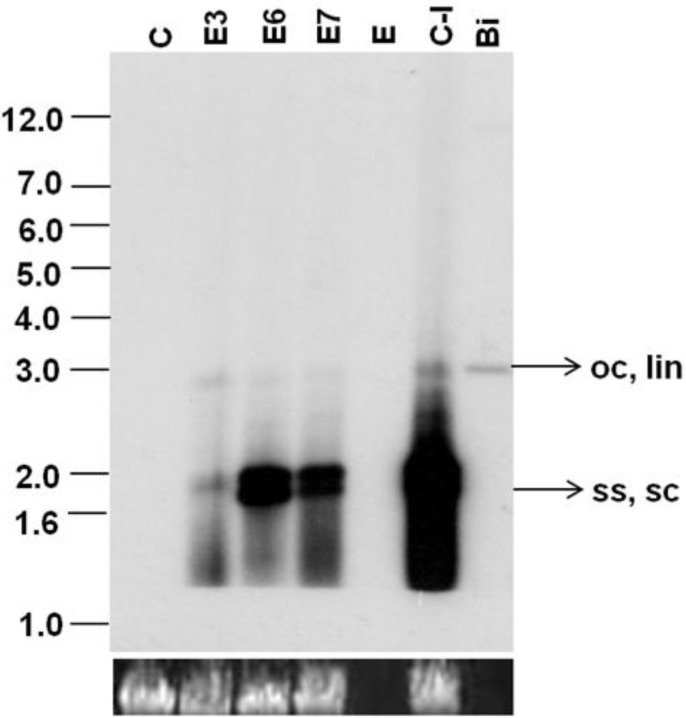
Southern blot analysis of transgenic *Nicotiana benthamiana* plants agroinoculated with SLCMV-[Attur2] DNA A and DNA B partial dimers. DNA (1 µg) from control uninfected (C), control agroinoculated (C-I), and the three agroinoculated transgenic plants (E3, E6, E7) was loaded in the respective lanes. Bi: SLCMV-[Attur2] full-length DNA A (50 pg) was used as a positive control. [α-^32^P]dCTP-labeled full-length SLCMV-[Attur2] DNA A was used as the probe. Single-stranded (ss), supercoiled (sc), open circular (oc) and linear (lin) forms of viral DNA are marked. E: empty lane. The bottom panel represents equal loading of plant DNA (1 µg) in all lanes.

The effect of the *virE2* gene on SLCMV DNA accumulation was analyzed by Southern blotting with the SLCMV DNA A probe. A high level of viral ssDNA accumulated in control plants infected with partial dimers of SLCMV DNA A and DNA B (Integrated density value (IDV)-214,785-100%). All three transgenic plants exhibited reduction in viral DNA in comparison to the control plant (Figure 6). The plant E3 with an IDV of 9,672 showed the maximum reduction of 95% in viral DNA level. Plant E6 (IDV-104,748) and E7 (IDV-54,530) exhibited reduction levels of 52% and 75%, respectively. Varying levels of reduction of viral symptoms and SLCMV DNA levels were observed in *virE2* transgenic plants in comparison to SLCMV-infected control plants. The levels of reduction in SLCMV DNA in the agroinoculated E3, E6 and E7 transgenic plants did not show a good correlation to the *vir*E2 transcript level in the transgenic plants (Figure 4).

## 4. Discussion

Several transgenic approaches based on viral and non-viral genes have been used to achieve geminivirus resistance [8]. We studied the efficacy of a non-viral protein, *A. tumefaciens* VirE2, to develop tolerance against SLCMV. VirE2 is a ssDNA binding protein [36] which binds to ssDNA in a cooperative manner and ensures that the complete ssDNA is coated with the protein. This protects the *Agrobacterium* T-strand from nuclease attack during the transfer process. VirE2 contains two bipartite nuclear localization signals and both are required for targeting VirE2 to the nucleus [37,38,39,40]. These properties of *virE2* prompted us to select the gene for engineering tolerance against SLCMV which has a ssDNA genome.

*Agrobacterium* VirE2 functions in the plant cell during T-DNA transfer. Citovsky *et al.* [35] showed that transgenic tobacco plants which expressed vir*E2* complemented a *virE2* mutation in *A. tumefaciens* and restored tumourigenesis. We found that all *virE2* transgenic plants E1, E2, E3, E5, E6 and E7, generated by us, complemented the *virE2* mutation in the *A. tumefaciens* strain A348mx358 and caused tumourigenesis of leaf discs in a hormone-minus medium (Figure 3). Thus, the VirE2 protein expressed in the transgenic plants is functional. The E3, E6 and E7 plants accumulated the *virE2* transcript. The three plants exhibited a reduction in disease symptoms when challenged by agroinoculation with the partial dimers of SLCMV–[Attur2] DNA A and DNA B. The level of SLCMV DNA was reduced to 95%, 52% and 75% in the transgenic plants E3, E6 and E7, respectively, in comparison to the SLCMV DNA levels in the control plants. In a previous report [22] we showed that leaf disc-agroinoculation of VirE2-expressing *N. tabacum* plants displayed a reduction in MYMV DNA levels. The MYMV agroinoculation experiments in the previous report [22] were limited to only leaf discs of *virE2* transgenic tobacco plants. Therefore, information was not generated on whether MYMV disease symptoms were reduced by *virE2* in the whole plants. In this report, the susceptibility of *N. benthamiana* plants to SLCMV permitted us to agroinoculate the whole *virE2* transgenic plants. The results clearly show that VirE2 reduced the SLCMV symptom level and brought down the SLCMV DNA accumulation in *virE2* transgenic *N. benthamiana* plants. In the current report, we have used *N. benthamiana* plants, rather than leaf discs, for agroinfection with SLCMV. These findings show that plant-expressed VirE2 reduces the levels of both MYMV and SLCMV and suggest that *virE2* may be useful for controlling geminiviruses in general and perhaps other DNA viruses as well.

Local and systemic spread of geminiviral DNA is essential to establish infection in different parts of a plant. In bipartite begomoviruses, nuclear shuttle protein (NSP) and movement protein (MP) play important roles in viral movement. NSP helps in the transport of geminivirus DNA from the nucleus to the cytoplasm, whereas MP facilitates the viral movement between the cells [41,42,43,44]. Two models, relay race model [44,45] and couple skating model [43,46], have been proposed for geminivirus movement. As per the couple skating model, the viral ssDNA bound to NSP shuttles between the nucleus and the cytoplasm. The complex then interacts with MP to cross the cell boundary. The *Tomato leaf curl virus* (ToLCV) genome modified to express the M13 phage ssDNA binding protein g5p [20] developed only mild symptoms and did not spread efficiently in *N. benthamiana* plants. Binding of g5p with the ssDNA of ToLCV may have competed with the NSP binding to ssDNA and thereby reduced the spread of the viral DNA. As in the case of g5p, the ssDNA binding protein VirE2 also might cooperatively bind to SLCMV ssDNA in the nucleus, thus preventing NSP binding and shuttling to the cytoplasm for cell to cell and systemic movement. It would very useful to study whether VirE2 can out-compete NSP in an *in vitro* ssDNA binding assay. Our reports show that *A. tumefaciens* VirE2, with the unique features of ssDNA binding and nuclear localization, is very effective in both MYMV [22] and SLCMV and holds promise to develop broad spectrum geminivirus tolerance.
